# Protein phosphatase 1 regulatory subunit 3G (PPP1R3G) correlates with poor prognosis and immune infiltration in lung adenocarcinoma

**DOI:** 10.1080/21655979.2021.1985817

**Published:** 2021-10-21

**Authors:** Xingli Zhuo, Lan Chen, Zongwei Lai, Jiansheng Liu, Shengjun Li, Ahu Hu, Yuansheng Lin

**Affiliations:** a Department of emergency and critical care medicine, The Affiliated Suzhou Science&Technology Town Hospital of Nanjing Medical University, Suzhou, China; b Department of Respiratory Medicine, Gannan Medical University, Ganzhou, China; c Department of Respiratory Medicine, Ganzhou People’s Hospital, Ganzhou, China

**Keywords:** PPP1R3G, lung adenocarcinoma, prognosis, immune, bioinformatics

## Abstract

The protein phosphatase 1 regulatory subunit 3 G (PPP1R3G) participates in many tumor biological processes; however, its effects on lung adenocarcinoma (LUAD) have not been clarified. Therefore, this study aimed to explore the correlation between PPP1R3G and the prognosis and immune invasion of LUAD. We evaluated the relationship between PPP1R3G and LUAD using a wide range of databases and analysis tools, including UALCAN, TIMER, miRDB, The Human Protein Atlas and the MethSurv database. First, we explored the mRNA and protein expression levels of PPP1R3G in LUAD, and results were validated using real-time PCR. Next, we explored the relationship between PPP1R3G expression and clinical features. Finally, Kaplan-Meier curves and Cox regression were employed to investigate the prognostic significance of PPP1R3G in LUAD. In addition, we explored the relationship between the expression of PPP1R3G and immune infiltration using the TIMER database. We analyzed the relationship between PPP1R3G and methylation using MethSurv database. Results showed that PPP1R3G expression in LUAD tissues was higher than that in normal tissues, and high expression was suggestive of a poor prognosis. Moreover, PPP1R3G expression was positively correlated with the immune infiltration of CD4 + T cells, macrophages, neutrophils, and dendritic cells. PPP1R3G copy number variations also demonstrated remarkable associations with the levels of B cells, CD4 + T cells, macrophages, neutrophils, and dendritic cells. Finally, a PPP1R3G-associated regulatory network was constructed. Overall, PPP1R3G might be a poor prognostic biomarker for LUAD and is associated with tumor immune cell infiltration.

**Abbreviations:** LUAD: Lung adenocarcinoma; PPP1R3G: The protein phosphatase 1 regulatory subunit 3G; OS: overall survival; CI: confidence interval; CNV: copy number variance; HR: Hazard Ratio; ROC: receiver operating characteristic curve; AUC: area under the curve; TCGA: The Cancer Genome Atlas

## Introduction

Lung cancer is one of the primary causes of cancer incidence and mortality worldwide, with approximately 2.2 million new cases of lung cancer and 1.8 million deaths in reporting 2020, accounting for approximately 1 in 5 (18.0%) cancer deaths [1]. Non-small cell lung cancer is the major subtype of lung cancer, and lung adenocarcinoma (LUAD) is the predominant subtype of this lung cancer [2]. A majority of patients (approx. 75%) present with advanced disease (stage III/IV) at diagnosis time and despite the remarkable advancements in the oncological management of late-stage lung cancer in recent years, the five-year survival rate remains poor at only 16% [3,4]. Consequently, identifying suitable biomarkers for prognosis of LUAD is very important.

PPP1R3G, a recently discovered G subunit, is correlated with the process of postprandial glucose and lipid homeostasis in the liver [5]. Daytime expression patterns of glycogen and glucose metabolism genes (Gck, Glut2, Gys2, Pklr and PPP1R3G) in the liver can be disrupted by chronic alcohol feeding. Studies have indicated that PPP1R3G is involved in maintaining the normal glycogen circadian rhythms in the liver [6]. Protein phosphatase 1 regulatory subunit 3 G (a Smad3 target) is a glucose metabolism-related enzyme that can be regulated by peroxisome proliferator-activated receptor gamma [7]. In 3T3L1 cells, high expression of PPP1R3G leads to elevated glycogen and triglyceride levels. Studies have shown that PPP1R3G provides an important link between glycogen metabolism and lipid metabolism in vivo [8]. Of note, PPP1R3G was also confirmed to be directly phosphorylated by serine-threonine protein kinase (AKT), and thus could mediate hepatic glycogen biosynthesis, as well as postprandial blood glucose clearance induced by insulin [9]. In addition, another study reported that the biological relationship between PPP1R3G and Sorcin could be related to the calcium, glucose, and lipid homeostasis [10]. A study also found that the PPP1R3G was downregulated after treatment with toxic Microcystis aeruginosa, suggesting that it plays a critical role in detoxification, along with the antitoxic mechanisms of microcystin in fish [11]. Researchers found that PPP1R3G was upregulated in transcripts after GCRV infection in grass carp, which indicated that PPP1R3G plays a negative role in anti-GCRV immune responses [12]. PPP1R3G plays a role in many diseases, but few studies have shown that PPP1R3G is associated with prognosis or immunity in LUAD.

Herein, This study aimed to investigate the prognostic value of PPP1R3G and its relationship with tumor immune cell infiltration in LUAD. Firstly, PPP1R3 expression level was compared between LUAD and nonmalignant lung tissues in the TCGA data resource. The expression of PPP1R3 protein was also explored in the CPTAC database. The association of PPP1R3G expression with clinicopathological features was explored in the TCGA data resource. Then, relationship of PPP1R3 expression with the prognosis of individuals with LUAD was explored via the Kaplan-Meier Plotter. Further, GO along with KEGG pathway enrichment analyses were employed to explore the prospective function of PPP1R3 in LUAD. Finally, TIMER was used to analyze the relationship between PPP1R3G and immune infiltration. We demonstrate for the first time that PPP1R3G significantly affects the prognosis of LUAD patients and is associated with tumor immune cell infiltration.

### Methods

#### Data source

LUAD mRNA normalized count data derived from the RNAseq Htseq platform were retrieved from TCGA (https://cancergenome.nih.gov/). Overall, 594 samples consisting of 535 cancer and 59 nonmalignant samples were obtained from LUAD-TCGA. This research was performed in compliance with tenets of the Helsinki International Conference and its Declaration.

#### Detecting differential expression of PPP1R3G

Data from the TCGA were employed to investigate the differences in PPP1R3G expression between nonmalignant and tumor tissues. Next, we assessed the differential PPP1R3G mRNA expression in different clinical parameters. The CPTAC data resource was used to assess the total protein expression of PPP1R3G in LUAD. The Human Protein Atlas database was used to analyze protein expression in LUAD and normal tissues via immunohistochemistry.

#### Analysis of prognostic potential

LUAD patients from the TCGA database were divided into two groups according to PPP1R3G expression. Overall survival (OS) was analyzed in the TCGA database using survival packages in the R software v3.6.1. According to clinical properties, PPP1R3G mRNA expression levels were compared in patients with LUAD by log2 calculation. Additionally, the UALCAN data resource (http://ualcan.path.uab.edu/index.html) [13] was employed to verify the OS of patients with TCGA-LUAD.

#### Immune infiltration analysis of PPP1R3G

TIMER (https://cistrome.shinyapps.io/timer/) [14] is a powerful database that can analyze the immune invasion levels of different tumors in the TCGA database. We evaluate the relationship between PPP1R3G expression and immune cell infiltration (B cells, CD4 T cells, CD8 T cells, neutrophils, macrophages, and dendritic cells) via gene module. We analyze the correlation between PPP1R3G gene somatic copy number alterations and tumor infiltration levels by SCNA module in LUAD.

#### DNA methylation analysis of PPP1R3G

A MethSurv tool was used to analyze the DNA methylation of PPP1R3G and explored the prognostic value of CpG sites in LUAD [15]. The relationship between PPP1R3G expression and DNA methylation was determined by Pearson’s tests.

#### Gene Enrichment Analysis Based on PPP1R3G Co-expressed Genes

We collected data on 59 genes that were positively associated with PPP1R3G using the UALCAN database. We performed GO and KEGG enrichment analyses based on 59 PPP1R3G co-expressed genes using Metascape. Next, we examined the target networks of miRNAs using the miRDB database. Cytoscape was then employed to visualize the miRNA-target of PPP1R3G in LUAD.

#### Establishment of the lung adenocarcinoma model in nude mice

Six-week-old nude mice were purchased from the Shanghai Jihui Experimental Animal Breeding Co., Ltd (China) (n = 3 in the LUAD group and paracancerous group). The 6-week-old mice were anesthetized by intraperitoneal injection of 45 mg/kg pentobarbital sodium, and then the co-precipitate of *AdCre:CaPi* (125 μL) [16] was dripped into the nasal cavity of the mice. This process was repeated in duplicate every 5 days for 42 days. The mice were sacrificed 42 days after induction. The mice were euthanized by CO2 euthanasia, at an initial CO2 flow of approximately 20% to 30% V/min, with the flow rate increased after loss of consciousness. The CO2 airflow was maintained for more than 1 min after clinical death. Finally, cervical dislocation was performed.

#### Quantitative real‑time PCR (RT-qPCR)

TRIzol reagent (Invitrogen, Thermo Fisher Scientific, US) was used to extract total RNA from mouse tissues, and RNA was reverse transcribed into cDNA using the M-MLV Reverse Transcriptase kit (Promega). SYBR Premix Ex Taq (TaKaRa) was used in the amplification process on an ABI StepOnePlus real-time PCR system (Applied Biosystems). β-Actin was used as an internal control. We analyzed the qRT-PCR results using the 2^−ΔΔCt^ method. The primers used were as follows: forward, 5ʹ-GGCAGTGTTCTCAGTGTT-3ʹ and reverse: 5ʹ-GTAAGGACCAAGTCTCAAGT-3ʹ.

#### Statistical analysis

Data were analyzed using the GraphPad Prism 8 software. Student’s t-test was used to analyze the data. Statistical significance was set at p < 0.05.

## Results

Both bioinformatics analysis and experimental verification revealed that the expression of PPP1R3G was significantly elevated in LUAD tissues. Overexpression of PPP1R3G was associated with poor prognosis of LUAD. We also found that PPP1R3G correlated with immune cell infiltration in LUAD.

### PPP1R3G was upregulated in LUAD

**Figure 1.**
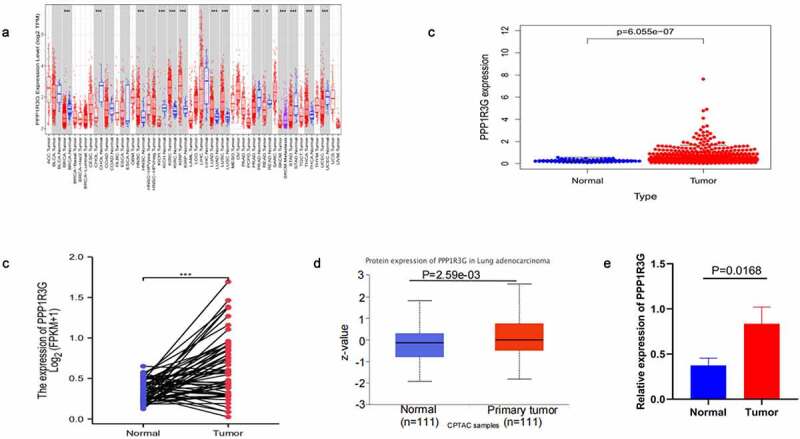
PPP1R3G was upregulated in lung adenocarcinoma (LUAD)

To explore PPP1R3G expression in diverse types of tumors, we analyzed PPP1R3G expression using the TIMER database. We found that PPP1R3G was upregulated in LUAD tissues compared to non-cancerous tissues (Figure 1(a)). Differences in PPP1R3G expression between LUAD and nonmalignant lung tissues were extracted from the TCGA data resource. We thus established that PPP1R3G expression levels were upregulated in LUAD tissues comapred to nonmalignant lung tissues (P = 6.055e−07, Figure 1(b)). In addition, the expression of PPP1R3G in the nonmalignant tissues (pre-disease) and LUAD groups (post-disease) was different. The PPP1R3G in the cancer tissues was increased significantly, suggesting that the expression of PPP1R3G was elevated during the development of LUAD (P < 0.001) as indicated in Figure 1(c). Next, the differences in PPP1R3G protein expression were explored between LUAD and nonmalignant tissues. The PPP1R3G protein content was remarkably higher in the LUAD tissues than that in nonmalignant tissues in the CPTAC data resource (P = 2.59e−03, Figure 1(d)). Finally, we used quantitative PCR to verify the expression level of PPP1R3G in normal tissues and lung adenocarcinoma tissues of mice. The relative mRNA expression level of PPP1R3G in LUAD tissues was higher than that in normal tissues (Figure 1(e), p = 0.0168), which was consistent with our analysis. However, we found protein expression levels of PPP1R3G in lung adenocarcinoma and normal tissues using the Human Protein Atlas database, but there was no significant difference in protein expression levels (Supplementary Figure s1). Taken together, these results suggested that PPP1R3G was upregulated in LUAD tissues compared to that in nonmalignant lung tissues.

(a) PPP1R3G expression levels in diverse kinds of tumors. Data was extracted from The Cancer Genome Atlas (TCGA) database by TIMER.(b) PPP1R3G expression in LUAD tissues compared to that in nonmalignant lung tissues using comparison of samples in TCGA database.(c) PPP1R3G expression pre-disease and post-disease in the same patient.(d) Differential expression of PPP1R3G protein between LUAD and nonmalignant lung tissues in the CPTAC data resource.(e) PPP1R3G expression levels in LUAD tissues and normal lung tissues of mice.

Promoter methylation is an important regulator of gene expression. As a consequence, we used the UALCAN analysis to explore PPP1R3G promoter methylation levels and its relationship with LUAD patients characteristics. Interestingly, we found that the PPP1R3G methylation level was significantly higher in LUAD tissues than in normal tissues, regardless of smoking status, stage, or nodal metastasis (Supplementary Figure s2). To further explore the mechanism of methylation, we analyzed the relationship between PPP1R3G and all CPG sites using the MethSurv database. The DNA methylation heatmap of PPP1R3G is shown in Supplementary Figure s3a. Among the 13 CpG sites, cg11574745 showed the highest levels of methylation. The prognostic value of all CpG sites was analyzed. cg11574745 and cg07637123 were significantly associated with OS in LUAD (Supplementary Figures s3B-3N). These results indicate that the methylation level of PPP1R3G in LUAD may be an important factor affecting the prognosis of LUAD.

### PPP1R3G transcription in subgroups of LUAD

**Figure 2.**
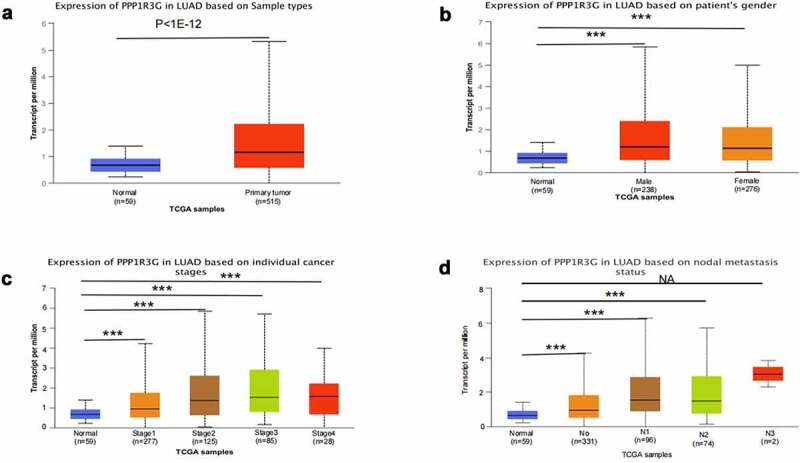
PPP1R3G mRNA expression in clinical characteristics of lung adenocarcinoma (LUAD) tissue

The expression of PPP1R3G mRNA in nonmalignant tissues (59 samples) and LUAD tumor tissues (515 samples) were analyzed according to the TCGA database. The results demonstrated that the level of PPP1R3 mRNA in LUAD was remarkably increased compared to that in nonmalignant tissues (P < 1e−12, Figure 2(a)). We subsequently investigated the association of PPP1R3G expression with the clinicopathological features of LUAD in the TCGA data. The results illustrated that PPP1R3G expression was remarkably linked to gender, clinical stage and node metastasis status (P < 0.001, Figure 2(b-d) and Table 1).

**Table 1.** Baseline charateristics of patients with LUAD

Characteristic	Low expression of PPP1R3G	High expression of PPP1R3G	p
n	267	268	
T stage, n (%)			0.076
T1	97 (18.2%)	78 (14.7%)	
T2	133 (25%)	156 (29.3%)	
T3	29 (5.5%)	20 (3.8%)	
T4	7 (1.3%)	12 (2.3%)	
N stage, n (%)			< 0.001
N0	196 (37.8%)	152 (29.3%)	
N1	36 (6.9%)	59 (11.4%)	
N2	26 (5%)	48 (9.2%)	
N3	0 (0%)	2 (0.4%)	
M stage, n (%)			0.225
M0	183 (47.4%)	178 (46.1%)	
M1	9 (2.3%)	16 (4.1%)	
Age, meidan (IQR)	66 (59, 72)	66 (59, 72)	0.862

(a) Differential expression of PPP1R3G mRNA between LUAD and nonmalignant lung tissues in the The Cancer Genome Atlas data resource.(b-d) Comparison of PPP1R3G mRNA expression based on patient’s gender, individual cancer, and nodal metastasis. NA P > 0.05, * P < 0.05; ** P < 0.01; *** P < 0.001.

### High PPP1R3G expression correlated with worse survival in LUAD patients

**Figure 3.**
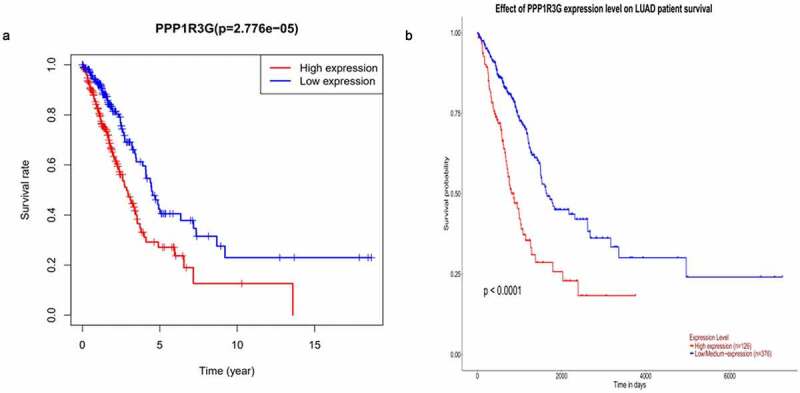
Prognostic function of PPP1R3G in lung adenocarcinoma

We found that elevated PPP1R3G expression was linked to poor OS in LUAD, while patients with low PPP1R3G expression levels had a longer OS in LUAD (P = 2.776e−05, Figure 3(a)). We subsequently evaluated the prognostic significance of PPP1R3G in LUAD using the UALCAN database and obtained similar results (P < 0.0001, Figure 3(b)). Based on these results, we speculated that the expression of PPP1R3G is significantly correlated with the progression and prognosis of LUAD.

(a) Overall survival analysis of PPP1R3G according to TCGA database.(b) Association of PPP1R3G expression with overall survival of lung adenocarcinoma patients from the UALCAN data resource.

### PPP1R3G is an independent factor affecting prognosis of LUAD

**Figure 4.**
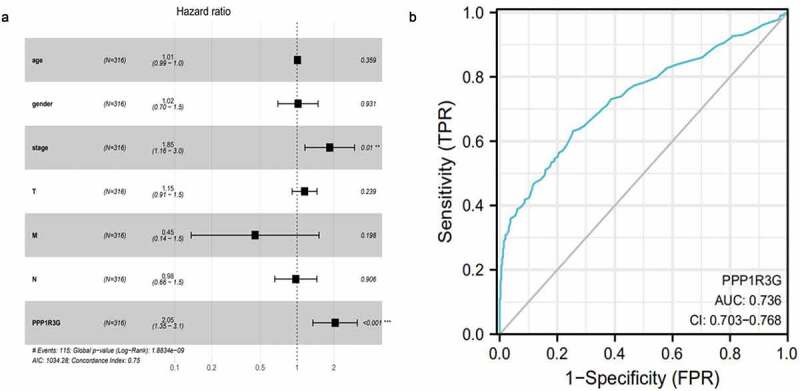
The independent prognostic factor and clinical diagnostic value of PPP1R3G in lung adenocarcinoma (LUAD)

In order to clarify factors that affected LUAD prognosis, Cox univariate analyses and Cox multivariate analyses were employed to compare clinical features of 316 individuals with LUAD. In the cox univariate analysis, the TNM stage (HR = 1.65, P = 2.58E-09, 95% CI: 1.40–1.95), T (tumor) (P = 8.60E-06, 95% CI: 1.32–2.02, HR = 1.63), N (node) (HR = 1.79, P = 2.41E-08, 95% CI: 1.46–2.20), and PPP1R3G expression (HR = 1.90, P = 4.17E-07, 95% CI: 1.48–2.43) were prognostic factors for LUAD. Next, we performed a cox multivariate analysis and found that for OS, TNM stage (HR = 1.85, P = 9.82E-03, 95% CI: 1.16–2.95), and PPP1R3G expression (HR = 2.05, 95% CI: 1.36–3.11, P = 7.86E-04) were prognostic parameters for LUAD, PPP1R3G was the most significant biomarker of all variables that were analyzed in OS (Figure 4(a) and Table 2, P = 7.86E-04, cox multivariate analysis). Furthermore, we constructed a receiver operating characteristic curve to explore the clinical diagnostic value of PPP1R3G in LUAD, and the results showed that the area under the curve (AUC) was 0.736 (Figure 4(b)). It was suggested that PPP1R3G has diagnostic value for the diagnosis of LUAD and PPP1R3G is an independent factor in the prognosis of LUAD.

**Table 2.** Univariate analysis and Multivariate analysis of the correlation of PPP1R3G expression with OS among lung adenocarcinoma

Parameter	Univariate analysis				Multivariate analysis			
	HR	HR.95 L	HR.95 H	pvalue	HR	HR.95 L	HR.95 H	pvalue
age	1.00	0.98	1.02	0.84	1.01	0.99	1.03	0.36
gender	1.04	0.72	1.49	0.85	1.02	0.70	1.49	0.93
stage	1.65	1.40	1.95	**2.58E-09**	1.85	1.16	2.95	**9.82E-3**
T	1.63	1.32	2.02	**8.60E-06**	1.16	0.91	1.46	0.24
M	1.76	0.96	3.20	0.07	0.45	0.13	1.14	0.20
N	1.79	1.46	2.20	**2.41E-08**	0.98	0.66	1.45	0.91
PPP1R3G	1.90	1.48	2.43	**4.17E-07**	2.05	1.36	3.11	**7.86E-04**

Bold values indicate p < 0.05.HR,hazard ratio; CI,confidence interval.

(a) Cox multivariate analysis was used to compare clinical characteristics of the forest plots of 316 patients with LUAD.(b) Receiver operating characteristic curve revealed the clinical diagnostic value of PPP1R3G in LUAD.

### PPP1R3G relates to immune infiltration level in LUAD

**Figure 5.**
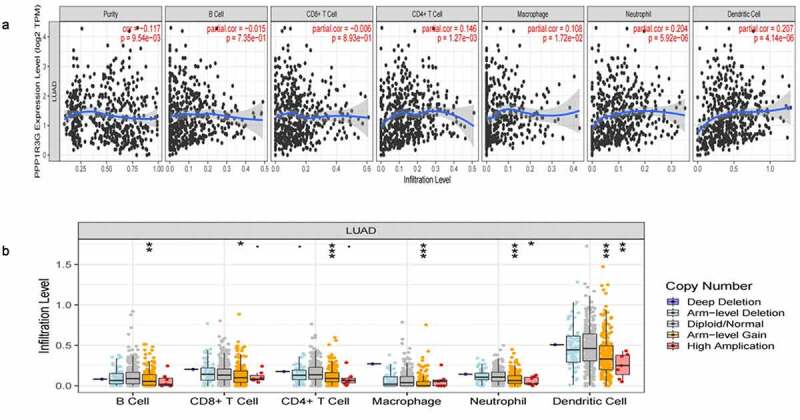
PPP1R3G relates to immune infiltration level in lung adenocarcinoma (LUAD)

To explore whether PPP1R3G was related to the immune invasion level in LUAD, we confirmed the relationship between PPP1R3G expression and immune infiltration level (Figure 5(a)). Interestingly, high PPP1R3G expression was associated with high immune infiltration levels of the immune cell populations, including CD4 + T cells (P = 1.27e-03, partial.cor = 0.146), macrophages (P = 1.72e-02, partial.cor = 0.108), neutrophils (P = 5.92e-06, partial.cor = 0.204) and dendritic cells (P = 4.14e-06, partial.cor = 0.207). We further compared the tumor infiltration levels in LUAD with different somatic copy number alterations in PPP1R3G (Figure 5(b)). Normal copy number or deletions spanning the PPP1R3G gene locus were correlated with increased immune cell infiltration. Our studies confirmed the important impact of PPP1R3G on immune infiltration level in LUAD.

(a) PPP1R3G expression was positively correlated with the infiltration levels of CD4 + T cells, macrophages, neutrophil, neutrophils, and dendritic cells in LUAD.(b) PPP1R3G CNV influences the infiltration level of B cells, CD4 + T cells, macrophages, neutrophils, and dendritic cells in LUAD.

### Functional annotation and pathway enrichment of PPP1R3G linked genes and the PPP1R3G regulatory network

**Figure 6.**
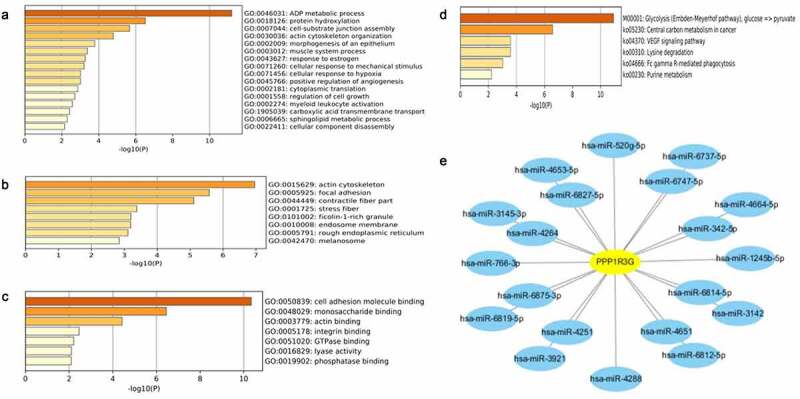
Gene enrichment analysis of PPP1R3G in lung adenocarcinoma cohorts

To explore co-expressed genes of PPP1R3G, we analyzed the mRNA sequencing data of LUAD patients according to the TCGA database. We found that the top 59 significant gene sets were positively correlated with PPP1R3G using the UALCAN database. To better understand the potential function of PPP1R3G, we performed Gene Ontology (GO) and Kyoto Encyclopedia of Genes and Genomes (KEGG) pathway analyses. In terms of biological processes, we found that the PPP1R3G associated genes were primarily enriched in protein hydroxylation, ADP metabolic process and positive regulation of angiogenesis (Figure 6(a)). Additionally, PPP1R3G was enriched in the actin cytoskeleton, focal adhesion, and contractile fiber cellular components (Figure 6(b)). Furthermore, we discovered that PPP1R3G was related to the molecular functions of cell adhesion molecule binding, monosaccharide binding and actin binding (Figure 6(c)). KEGG pathway analysis revealed enrichment in glycolysis, central carbon metabolism in cancer, and VEGF signaling pathway (Figure 6(d)). Next, we predicted the miRNA-target of PPP1R3G using miRDB. The data revealed 20 miRNA-targets of PPP1R3G, as shown in Figure 5(e). These data suggested the promising role of PPP1R3G in modulating tumor progression in LUAD patients, and the results might show a potential regulatory network of PPP1R3G in LUAD.

(a-c) Enriched GO terms of PPP1R3G-linked genes.(d). Enriched KEGG cascades of PPP1R3G-related genes.(e).The PPP1R3G-miRNA regulatory network.

## Discussion

Lung cancer remains the primary cause of cancer-mediated fatalities globally, with its five-year survival having remaining relatively poor [13]. Because of the poor prognosis documented in the latest cancer report released in 2021, exploring the precise molecular mechanisms of LUAD may enhance the discovery of new biomarkers and therapeutic targets, which is necessary to enhance the prognosis of individuals with LUAD [17,18].

Herein, we can discovered that PPP1R3G expression was remarkably elevated in LUAD tissues compared to that in the nonmalignant tissues (Figure 1(b)). Furthermore, in contrast to pre-disease, PPP1R3G is highly expressed post-disease (Figure 1(c)), suggesting that PPP1R3G is remarkably linked to LUAD onset and progression. Next, we explored the association of PPP1R3G with clinicopathological features of individuals with LUAD in the TCGA data resource to elucidate possible functions of PPP1R3G in lung cancer. The results revealed that PPP1R3G expression was significantly associated with gender, clinical stage, and node metastasis status. In addition, Kaplan-Meier Plotter and UALCAN data resources were employed to explore LUAD prognosis, and the data suggested that elevated PPP1R3G expression was remarkably linked to worse OS. In summary, our data illustrated that PPP1R3G could be a prospective prediction and prognostic biomarker of LUAD. Nevertheless, further studies should be conducted to explore the biological functions of PPP1R3G in LUAD.

PP1 plays a vital role in numerous cellular processes, some of which are correlated to centrosome biology, including mitosis, cytokinesis, and cell cycle [19–21]. The PP1A holoenzyme consists of a catalytic subunit and a modulatory subunit, which regulate the physiological roles of PP1 by modulating enzymatic activity along with specificity [22]. PPP1R3, a corresponding regulatory subunit 3 of PP1, is located on chromosome 7q31.1-q31.2, encoding a polypeptide composed of 1122 amino acids [23]. Loss of heterozygosity at 7q31 has been reported to occur in multiple types of human cancers, including lung carcinomas, breast, hematological, stomach, ovary, colon, and kidney malignancies [24–26]. Kohno et al. also found that nonsense and missense mutations of the PPP1R3 are common in primary non-small cell lung carcinomas, ovarian carcinoma, gastric carcinoma, and colorectal carcinoma [22]. These results indicate that PPP1R3 could serve as a tumor repressor gene in human cancers. PPP1R3G, a member of the PPP1R3 family of proteins, is a modulatory subunit of PP1, and plays a critical role in the modulation of postprandial glucose homeostasis during the fasting-feeding transition through its regulation of liver glycogenesis [27]. Herein, we found that PPP1R3G was upregulated in LUAD, and higher PPP1R3G expression resulted in worse survival. On the basis of our research, we speculate that PPP1R3G contributes to the process of LUAD by disturbing the intracellular signaling pathway by PPl.

To clarify molecular mechanisms responsible for the function of PPP1R3G in LUAD, we analyzed the correlation between PPP1R3G expression and immune infiltration in LUAD. Our results suggested that PPP1R3G expression has a remarkable positive relationship with the infiltration levels of B cells, CD4 + T cells, and macrophages in LUAD (Figure 4(a)). These results suggest that high PPP1R3G expression correlates with immune infiltration in the tumor microenvironment, which itself correlates with the prognosis of small cell lung cancer [28]. The subset of patients with colorectal cancer with high immune infiltration have a poor prognosis; Their tumors had high levels of T cell infiltration and also showed increased expression of programmed death ligand 1 [29]. Although several studies have supported the point that immune cell infiltration leads to a poor prognosis, whether our results of our study are consistent with them requires further study. Then, in the inflammatory reaction, immune cells synthesize and secrete angiogenic factors to promote angiogenesis. Immune cells cooperate with stromal cells and malignant cells to stimulate endothelial cell proliferation and angiogenesis [30]. B7-H3, an immune checkpoint molecule, plays critical roles in proliferation, metastasis and tumorigenesis in diverse tumors. B7-H3 promotes angiogenesis in vitro [31]. Overall, angiogenesis and immune infiltration are closely related. Therefore, PPP1R3G may regulate immune infiltration through angiogenesis.

We also investigated the role of PPP1R3G and performed the GO and KEGG analyses. Most of the GO and KEGG categories were enriched in the ADP metabolic process and glycolysis processes. miRNAs are core modulators of genes which function at the post-transcriptional levels [32,33]. We subsequently established a PPP1R3G-miRNA regulatory network using miRDB, which is potentially valuable for investigating PPP1R3G modulatory systems given the integration of prior knowledge.

Herein, we illustrated that elevated PPP1R3G content in LUAD was remarkably linked to worse OS and could be associated with the PPl signaling cascade. However, this study had several limitations: First, a larger patient sample size of LUAD is required to validate the prognostic significance of PPP1R3G. Further, Due to the database, we were unable to further explore the relationship between PPP1R3G and tumor immunocytes. we can only explore the possible mechanism in the future Work.

## Conclusions

PPP1R3G might be a poor prognostic biomarker for LUAD and is associated with tumor immune cell infiltration.

## Supplementary Material

Supplemental Material

## Highlights

1. High expression of PPP1R3G indicates poor prognosis in LUAD patients.
2. PPP1R3G correlates with prognosis and immune infiltration in lung adenocarcinoma
3. PPP1R3G may be a prognostic biomarker of LUAD and may be a new therapeutic target.
