# Supplementary material for: Protein phosphatase 1 regulatory subunit 3G (PPP1R3G) correlates with poor prognosis and immune infiltration in lung adenocarcinoma
Source: Bioengineered. 2021 Oct 21;12(1):8336–46. doi: 10.1080/21655979.2021.1985817 (PMC8806970; doi:10.1080/21655979.2021.1985817)
Supplement: Supplemental Material [file KBIE_A_1985817_SM9433.zip › supplementary/Supplementary Figure legend.docx]

**Supplementary Figure 1. Protein expression level of PPP1R3G in lung adenocarcinoma.**

Immunohistochemical (IHC) staining of PPP1R3G expression in lung adenocarcinoma samples and normal tissues from the Human Protein Atlas.

**Supplementary Figure 2. PPP1R3G promoter methylation level in lung adenocarcinoma (LUAD).**

A.The methylation levels of PPP1R3G in LUAD tissues compared to that in non-malignant tissues according to TCGA data resource.

B-D. Comparison of methylation levels of PPP1R3G based on smoking status, stage and nodal metastasis. NA P>0.05, * P < 0.05; ** P < 0.01; *** P < 0.001.

**Supplementary Figure 3. The heatmap of PPP1RG DNA methylation and the prognostic value of CpG sites.**

A. The heatmap of methylation levels of PPP1R3G. Heat map methylation level (1 = complete methylation; 0 = completely unmethylated) is shown as a continuous variable from blue to red.

B-N. Survival analysis of all methylation probes; P < 0.05 was considered statistically significant.
