# Supplementary figures and images for: Protein phosphatase 1 regulatory subunit 3G (PPP1R3G) correlates with poor prognosis and immune infiltration in lung adenocarcinoma
Source: Bioengineered. 2021 Oct 21;12(1):8336–46. doi: 10.1080/21655979.2021.1985817 (PMC8806970; doi:10.1080/21655979.2021.1985817)

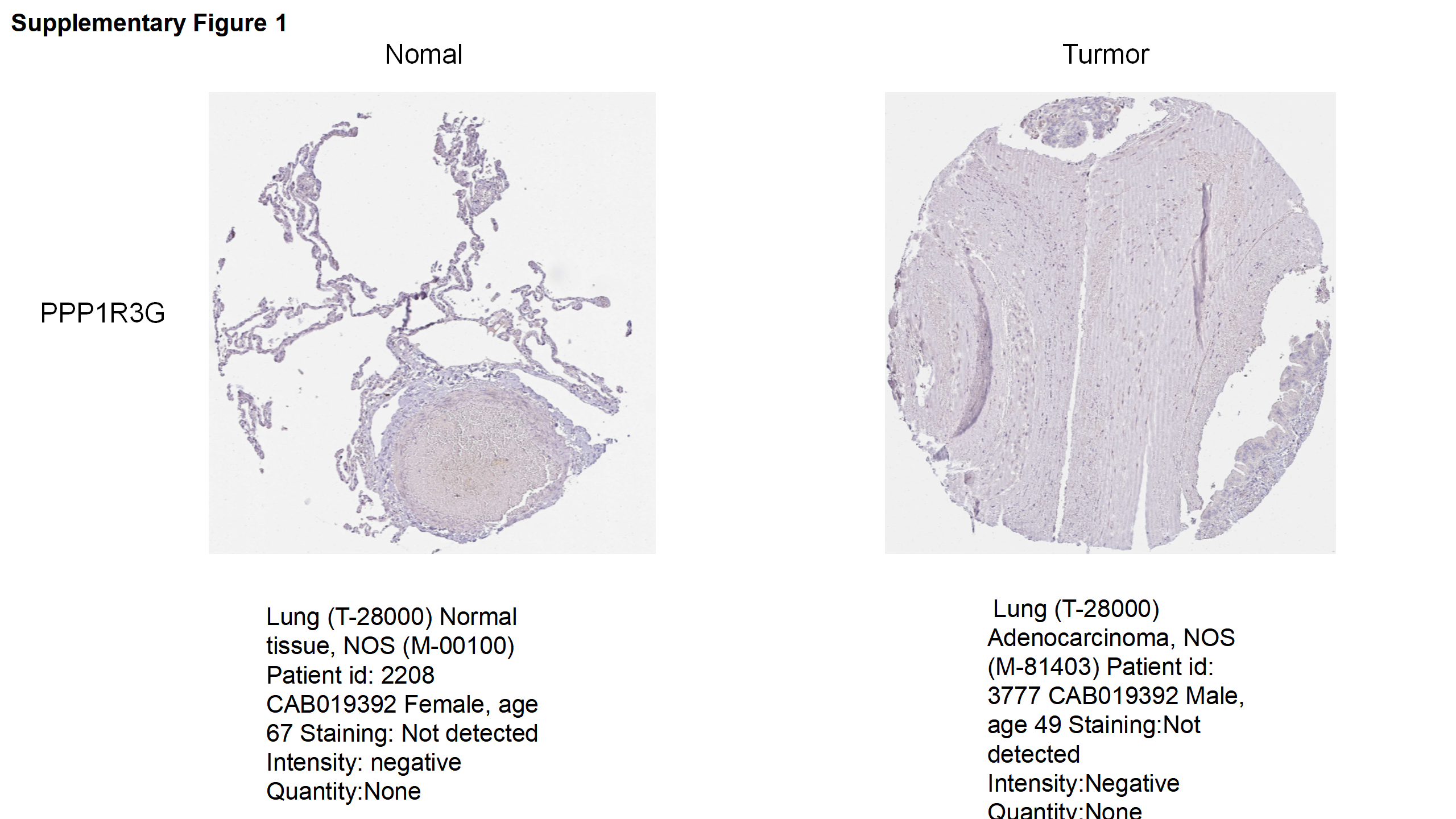

Supplement: Supplemental Material [file KBIE_A_1985817_SM9433.zip › supplementary/Supplementary Figure 1.png]

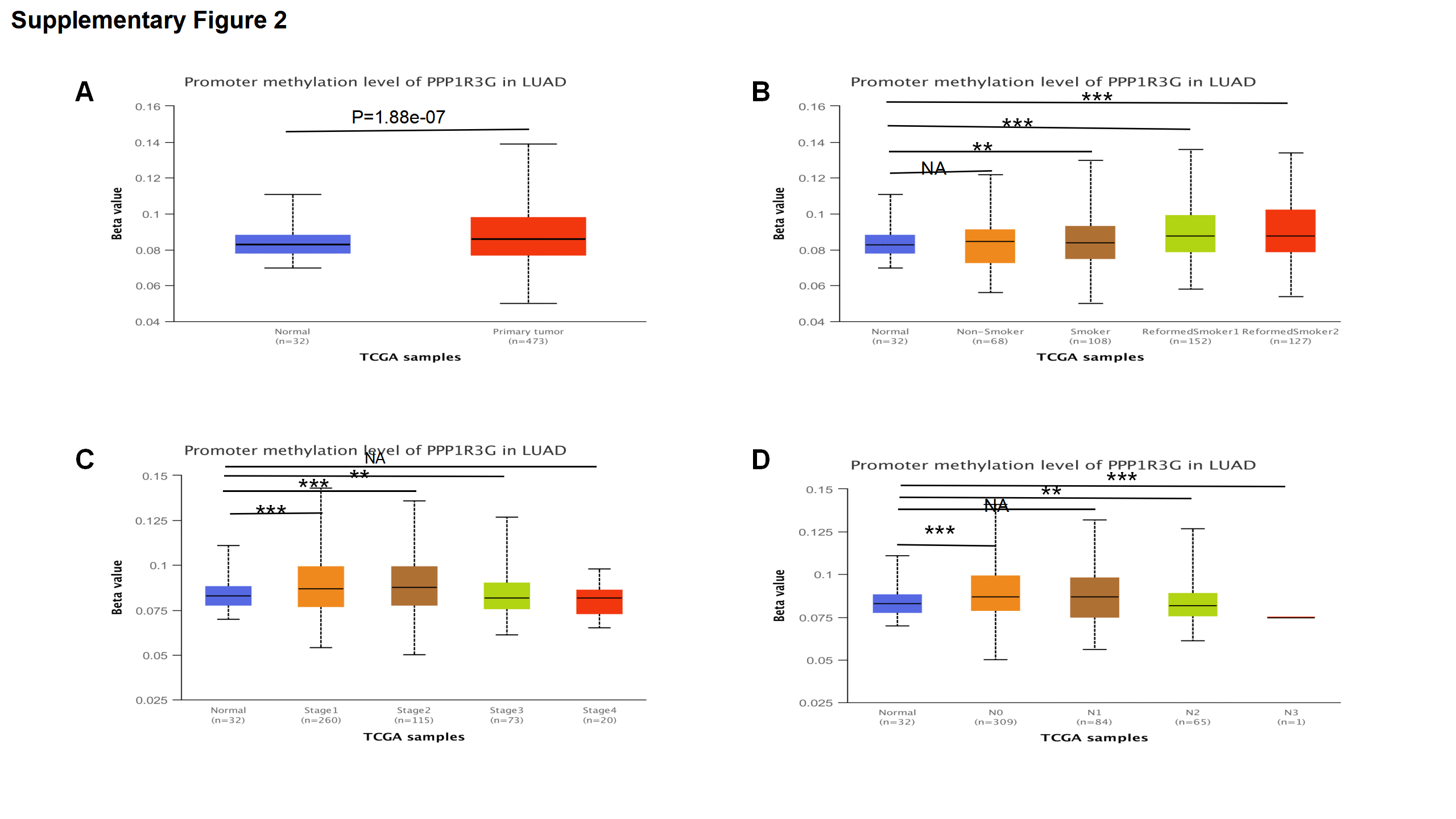

Supplement: Supplemental Material [file KBIE_A_1985817_SM9433.zip › supplementary/Supplementary Figure 2.png]

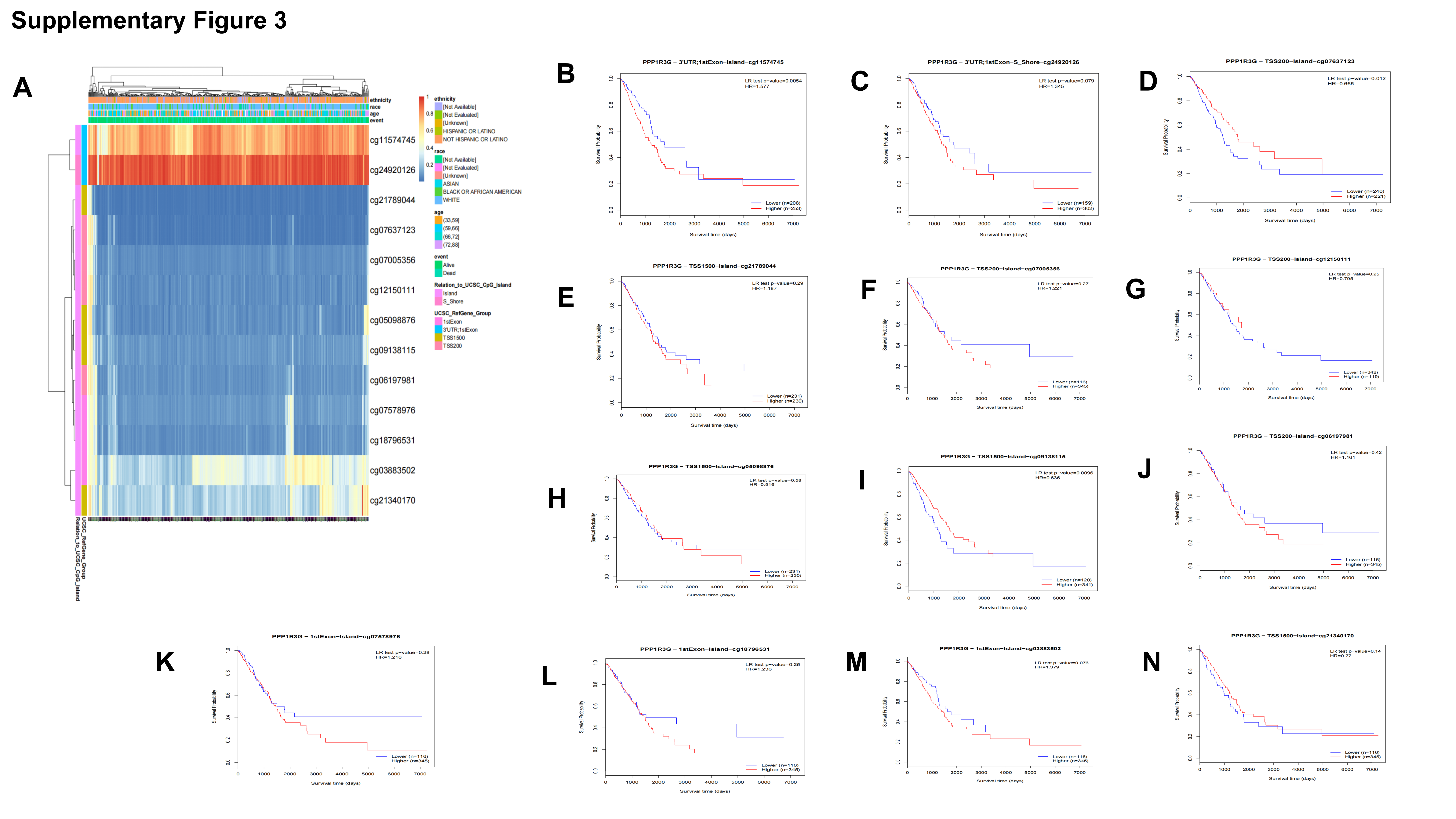

Supplement: Supplemental Material [file KBIE_A_1985817_SM9433.zip › supplementary/Supplementary Figure 3.png]
